# Investigating user perceptions of commercial virtual assistants: A qualitative study

**DOI:** 10.3389/fpsyg.2022.944714

**Published:** 2022-09-06

**Authors:** Leilasadat Mirghaderi, Monika Sziron, Elisabeth Hildt

**Affiliations:** Illinois Institute of Technology, Chicago, IL, United States

**Keywords:** virtual assistants, human-machine communication, human-technology interaction, anthropomorphism, information communication technology, commercial virtual assistants

## Abstract

As commercial virtual assistants become an integrated part of almost every smart device that we use on a daily basis, including but not limited to smartphones, speakers, personal computers, watches, TVs, and TV sticks, there are pressing questions that call for the study of how participants perceive commercial virtual assistants and what relational roles they assign to them. Furthermore, it is crucial to study which characteristics of commercial virtual assistants (both existing ones and those envisioned for the future) are perceived as important for establishing affective interaction with commercial virtual assistants. By conducting 26 interviews and performing content analysis of the interview transcripts, this study investigates how the participants in the study perceive, engage, and interact with a variety of commercial virtual assistants. The results lead to better understanding of whether forms of attachment are established or if some sort of relationship is produced between humans and commercial virtual assistants. Key takeaways from our results indicate that, in their current state, the lack of humanlike characteristics in commercial virtual assistants prevents users from forming an emotional attachment to commercial virtual assistants, but this does not deter them from using anthropomorphic language to describe commercial virtual assistants. Yet, our results reveal that users expect commercial virtual assistants’ attributes to be more humanlike in the future.

## Introduction

There is no shortage of science fiction books, movies, and TV shows that proliferate various conceptions of the future of human-virtual assistant relationships. In the movie Her^1^ a lonely man becomes emotionally attached to his virtual assistant. In the film Jexi,^2^ the roles are reversed as a self-aware virtual assistant becomes emotionally attached to its owner. Furthermore, some commercials such as Amazon Alexa ads,^3^ and the song “True Romance” by “Die Ärzte” have addressed attachment to virtual assistants. Similar to the other advancements in the technology, it might not be before long that such fictional communications become more possible than ever. Therefore, there is a need to investigate how such HMCs are currently being perceived and what are users’ expectation for the future of this type of communications.

Interdisciplinary literature on the social and ethical implications of technology, artificial intelligence (AI), and human-technology interaction has suggested attachments to technology, including smartphones and virtual assistants. In the context of interaction with information communication technologies (ICTs), research regarding the relationships between humans and technology is significant. The way humans use and interact with technology illustrates the roles of technology in their lives, and how they think about technology can define what roles they attribute to it. The tendency to ascribe human-like behaviors, or to ascribe social roles, to technology has been interpreted as playing a central role in several theoretical approaches to technology ethics and roboethics (Coeckelbergh, 2018; Gunkel, 2018; Danaher, 2020). Compared with other forms of technology, commercial virtual assistants are one of the most widely available technologies that are designed to mimic human-like behaviors and take social roles. Therefore, there is a need to investigate how users perceive commercial virtual assistants. In this study, we investigated such perceptions by performing content analysis on the transcribed interviews.

In order to investigate the user perceptions of commercial virtual assistants, our study began with four primary research questions: How do study participants respond to and interact with commercial virtual assistants like Siri, and Alexa? What can be concluded about study participants being attached (emotionally or otherwise) to commercial virtual assistants? What social roles are attributed to commercial virtual assistants? Are they considered a playfellow? What are the expectations for future interaction with commercial virtual assistants and future (social) roles of commercial virtual assistants?

Results from our study highlight human perceptions of AI systems in the form of commercial virtual assistants. Our study addresses how participants use gendered pronouns to describe their interactions with commercial virtual assistants. By asking participants questions like “Do you think your Virtual Assistant is aware of and responsive to your intentions, actions, and feelings?” and “Do you think Virtual Assistants can understand you?” our paper touches on human perceptions of commercial virtual assistant agency. Our results also share insights into trust and AI systems. Our participants were asked “Do you trust the information you receive from your AI Virtual Assistant? Which information do you trust giving your Virtual Assistant?” Our study explicitly addresses the cultural implications of non-native English speakers using Western AI systems by asking “Is there a language barrier that prevents you from using Virtual Assistants?” Other perceptions highlighted in our study include, frequently used tasks and features of commercial virtual assistants, spatial awareness when using commercial virtual assistants, whether commercial virtual assistants can be perceived as playfellows and companions, whether emotional attachment is perceived when using commercial virtual assistants, and perceptions of commercial virtual assistants in comparison to human beings. Our results present a snapshot of the overlay of societal constructs onto commercial virtual assistant AI systems and the tip of the iceberg of when it comes to the interaction of humans and AI in the form of commercial virtual assistants. We hope that our research becomes a part of the “symbiotic relationship between efforts in communication research and SDS (spoken dialogue systems) development” (Gunkel, 2020, p. 155).

The rest of this paper is organized as follows. First, in order to provide a background and support the research questions, a literature review section is presented. Then, in the methods section, the participants in the study, the dataset, and the process of the data analysis are brought. The results are presented in section “Results” and the discussion and study limitations are presented in section “Discussion”. Finally, section “Conclusion” concludes this paper.

## Literature review

In the context of this paper, one of the most important concepts that is receiving a lot of attention recently in the literature is the human-machine interaction. Regarding human-machine interaction, several authors have put forward a “relational turn” framework (Coeckelbergh, 2018; Gunkel, 2018) or an “ethical behaviorism” framework (Danaher, 2020). Relational turn is associated with changing the focus from what properties the other has, to what takes place in the relationship itself (Coeckelbergh, 2018; Gunkel, 2018). Also, by emphasizing the behavior as “the primary and most important source of knowledge about the moral status of others,” ethical behaviorism takes a comparative approach and in the case of robots states that “If a robot looks and acts like a being to whom moral status is afforded then it should be afforded the same moral status, irrespective of what it is made from or how it was designed/manufactured” (Danaher, 2020, p. 2047). Such frameworks raise the research questions in this study regarding how participants perceive commercial virtual assistants, what social roles they assign to them, and whether they can form an attachment to these commercial virtual assistants. Furthermore, in such interactions, anthropomorphism is an important concept that needs to be considered in more detail as these agents are becoming more and more responsive to human inputs.

Anthropomorphism can be defined as “the attribution of distinctively human-like feelings, mental states, and behavioral characteristics to inanimate objects, animals, and in general to natural phenomena and supernatural entities” (Salles et al., 2020, p. 89). However, Koike and Loughnan (2021) acknowledge the limitation of the current social psychology models in addressing the complexities of our relations with virtual agents. Similarly, Gambino et al. (2020) propose an extension of “computers are social actors framework” (CASA) to account for the recent changes in the interaction of people and technologies. Therefore, there is a need for more studies to tackle the challenges associated with human and virtual agents’ communication. Also, since commercial virtual assistants are becoming more and more human-like, there is a need to study the future perception and expectations of users from this technology. Considering the current limitations of the social psychology models and the level of advancements in commercial virtual assistants, the research question regarding the future expectations of virtual assistants was raised. Also, in order to narrow down the scope of this study, there was a need for a clear definition of commercial virtual assistants to distinguish them from other types of virtual assistants such as embodied agents.

Embodied agents are characters that are controlled by computer programs. Traditionally, research studies on embodied agents were focused on agents that were generated and controlled by computers and were visualized using a monitor or projector (Norouzi et al., 2020). However, with the advancements in the technology, embodied agents became available in new forms of media; first with virtual reality, and then with augmented reality (Norouzi et al., 2020). While our study is not focused on embodied agents, it is worth noting that based on the systematic literature review of 50 research papers from 2000 to 2020, Norouzi et al. (2020) have identified assistive and companion roles as the emerging and future trends in this field. Such roles are analyzed in this paper.

Commercial virtual assistants in this study are not robots, not physically or virtually embodied AI—even though voice can also be considered an embodied feature (Pradhan et al., 2019). However, voice has an important role in communication and human-machine communication (HMC) is no exception. Furthermore, HMC may even be more complex than human-human interactions as according to Guzman (2019) “people are simultaneously interacting with multiple human and technological ‘layers,’ that can be perceived as sources (i.e., programmers, applications, hardware, interfaces)” (p. 344). When humans interact with a commercial virtual assistant, they are interacting with a variety of AI systems and AI activity. A basic interpretation of the AI involved in commercial virtual assistants includes, first implementing automatic speech recognition from the audio input from the user, then dialogue management of the audio input to determine what the user needs, and finally, to create a deliverable back to the user, text-to-speech synthesis. Natural language processing, natural language understanding, and natural language generation are included in more detailed interpretations of virtual assistant technology (Hirschberg and Manning, 2015). We use the term “commercial virtual assistant,” and consider the definition of this term as having the following main characteristics: (1). It is an application running on a remote computer. (2). Through an interface, it can receive input information (text and voice) from the user and provides a responsive action according to the provided input. (3). It enables the user to automate some predefined functions related to accessing information (Cooper et al., 2008, 2011). While others have used terms like “AI assistants”; “Artificially Intelligent Personal Assistants” (Danaher, 2018), “voice-based conversational agents”; “Personal digital assistants”; or “voice assistants” (Pradhan et al., 2019), the advantage of the term “commercial virtual assistant” is its neutrality. For instance, in our analysis we do not focus only on the importance of “voice,” of which “voice assistant” suggests. The term “commercial virtual assistant” does not overemphasize one trait of these AI systems over another.

Commercial virtual assistants in our study are differentiated from the technology investigated in relatively similar research studies. First, commercial virtual assistants are not physically embodied and are different from social robots (Scheutz, 2011; Darling, 2016). Second, commercial virtual assistants are not confined to a single device such as a voice-activated wireless speaker (Gao et al., 2018). Gao et al. (2018) have performed a content analysis on the reviews found on the Amazon Echo web page and performed several analyses, including personification and emotion analysis, in their opinion mining efforts. Their results illustrate that “30% of customers would like to treat Amazon echo as a human character because of its personified name (Alexa) and the ability to talk” (Gao et al., 2018, p. 9). While scholars such as Gao et al. (2018) have performed research on the reviews found online, in our study, we found the need to directly interview participants for their opinion through interviews to get a qualitative dataset for our analysis. Based on the survey of 247 papers from the International Conference on Intelligent Virtual Agents from 2000 to 2015, Norouzi et al. (2018) found that only 6.9% of the studies used qualitative data (i.e., from focus groups or interviews). Moreover, we did not confine our study to a single device or commercial virtual assistant.

The commercial virtual assistants in this study can be accessed from a variety of devices including, but not limited to, personal computers, cellphones, speakers, and watches. Our study focuses on the mobile application of AI enabled commercial virtual assistants due to the fact that these are currently the most common and accessible forms of interaction with virtual assistants or as Guzman (2019, p. 344) puts it, they are “routine part[s] of people’s daily lives as they are readily located in back pockets and purses.” For the sake of brevity and avoiding repetition, the term virtual assistants is used as a replacement for commercial virtual assistants.

## Materials and methods

We interviewed 26 students (graduate and undergraduate) from Illinois Institute of Technology between November 2019 and March 2020. The in-person interviews were based on two sets of interview questions: frequent users and former users of virtual assistants (see Appendix). We sought to find out how users interact with virtual assistants, whether they develop an attachment to virtual assistants, and if so, what reasons they give. We were also interested in whether users considered interaction with virtual assistants as a replacement for interaction with humans. To this end, we designed and conducted interviews based on the guidelines and principles of episodic interviewing (Flick, 2000). Episodic interviewing allows “study on the social representation of technological change in everyday life” (p. 75). We found this framework to be a good fit for studying virtual assistants as technologies with direct impacts on our daily lives. We were able to gather the narratives of the interviewees as social science data. The study was approved by the Illinois Institute of Technology Institutional Review Board.

### Participants

Illinois Institute of Technology students (both graduate and undergraduate) were notified about this research project through flyers, campus news, and social media platforms such as Facebook and Twitter. There were 26 participants (11 female, 15 male) between 18 and 54 years old, with an average age of 24.92 years (SD = 7.70). One interviewee did not disclose age. Furthermore, 11 (42.3%) of the participants identified themselves as internationals and 15 (57.7%) identified as native English speakers. Two sets of interview questions were prepared for frequent users (24 participants) and former users (2 participants) of virtual assistants. Except for the former users, all frequent users were currently using one or several virtual assistants through different devices such as their smartphones, smart speakers, or their personal computers. The most utilized virtual assistants in our study include Google Assistant (15 participants), Siri (14 participants), and Amazon Alexa (6 participants). 12 out of the 26 participants had experience with more than one virtual assistant.

### Materials and process

Interviews were held, and voice-recorded, in private after the participants gave their voluntary and informed written consent. Questions in the interview question sets for frequent users and former users were categorized into the following: General, Communicational, Emotional, and Demographic. Aside from general and demographic questions, questions were open-ended, identifying the ease of use, ease of communication, language barriers, trust, agency, attachment, and relationships associated with virtual assistants. The two sets of interview questions, for frequent users and former users can be found in Appendix. Both interview question sets had four categories of General, Communicational, Emotional, and Demographic to allow for the identification of themes. While the Demographic questions were the same in both sets, the other three categories in the Frequent Users set were designed to provide information about how the interviewees are currently interacting with their virtual assistants, while these categories in the Former Users set were designed to provide information about why they do not use virtual assistants or how they perceive others use of virtual assistants.

### Data analysis

After anonymized transcription, the interviews were coded using content analysis. By employing a systematic classification, content analysis was used for reducing the complexity of the large collection of texts and we were able to turn each transcribed and anonymized interview into a “short description of some of its features” (Bauer, 2000, p. 133, Frey et al., 2000). First, all research team members were familiarized with the data by reviewing the transcripts. Second, a codebook and categories were developed and finalized upon the agreement of all research team members. The codes were developed, and coding was performed following the principles of abductive analysis which is positioned between observation (induction) and rules (deduction) (Timmermans and Tavory, 2012). Table 1 provides a summarized version of the codebook. Third, each research team member individually performed coding of the interviews according to the codebook. Fourth, based on the agreement of all research team members, seven common themes (both prescribed in the questionnaire and independent from the questionnaire) were extracted that are discussed in the next section.

**TABLE 1:** Summarized codebook.

Types of VAs
Usage frequency
Tasks
Language barriers
Alone vs. In public
Understanding
Trust
Advantages/Disadvantages
Feelings
Playfellow/Friend
Disclosure
Emotional attachments
Loneliness
Gendered pronouns
Science fiction references
Future expectations
Age
Type of student
Language
Gender

## Results

A total of seven common themes were extracted and coded from transcribed interviews. Six themes were directly derived from the interview questions: features and tasks, spatial awareness, playfellow, companionship, trust, and perceived emotional attachment. The seventh theme, comparison with human beings, was identified after researchers familiarized themselves with the gathered data.

Questions regarding tasks and features identified the different tasks participants assign to their virtual assistants (here on referred to as VAs). Questions regarding spatial awareness were used to determine whether participants prefer to use their VAs in public or private spaces. Questions regarding playfellow investigated the possibility of VAs becoming a playfellow for the participants. Questions regarding companionship were used to help identify whether VAs help with alleviating feelings of loneliness. The possibility of forming an emotional attachment to VAs was investigated. The comparison with human beings theme was introduced as interviewees began to identify how VAs resemble, or differ from human beings, in terms of performing humanlike capabilities.

It should be mentioned that all quotations are pulled from the transcripts of frequent users. Also, quotations from interviewees are organized by interviewee number and frequency. The letter next to the interviewee number is in the alphabetic order and represents how many times a quotation is pulled from a certain interviewee. This is placed to ensure that the number of quotations from of a specific interviewee is controlled. For clarity, the quotation labeled #1a is a quotation from interviewee number one and the first instance/use of their quotation in the text, #8b is a quotation from interviewee number eight and the second instance/use of their quotation in the text.

### Features and tasks

**FIGURE 1:**
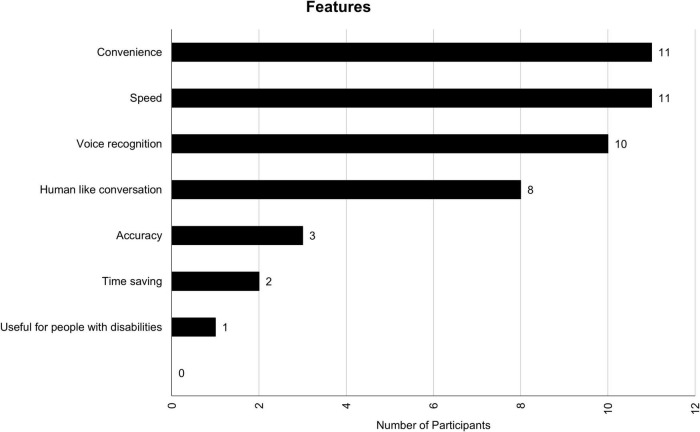
Features participants find useful in their VA.

**FIGURE 2:**
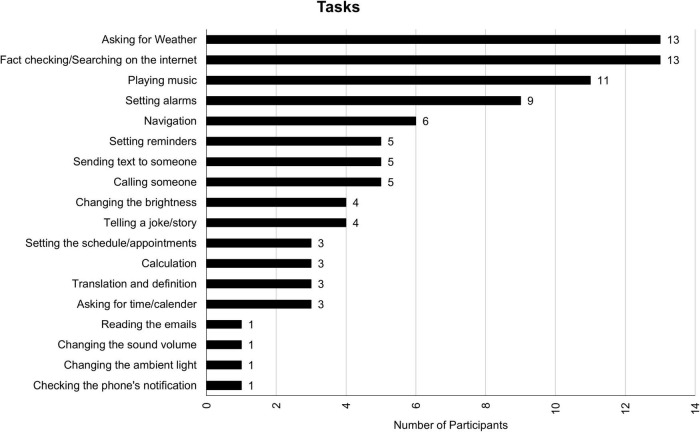
Tasks participants assign to their VA.

Participants use their VAs for a variety of tasks and find a variety of features useful. Figures 1, 2 contain the distribution for features and tasks, respectively. Among the features, handsfree features were mentioned most often. Handsfree features help accomplish a wide range of tasks including, but not limited to, looking for the weather, sending a text, and/or playing a song. A complete list of tasks can be found in Figure 2. Participants said they mostly use VAs for tasks that require picking up the phone, going to their desired application, and typing a command. In other words, they mostly use VAs as a replacement for physical labor. There were also instances of human-like conversations mentioned such as asking their VA to tell a joke, a bedtime story, and asking personal questions.

### Spatial awareness

**FIGURE 3:**
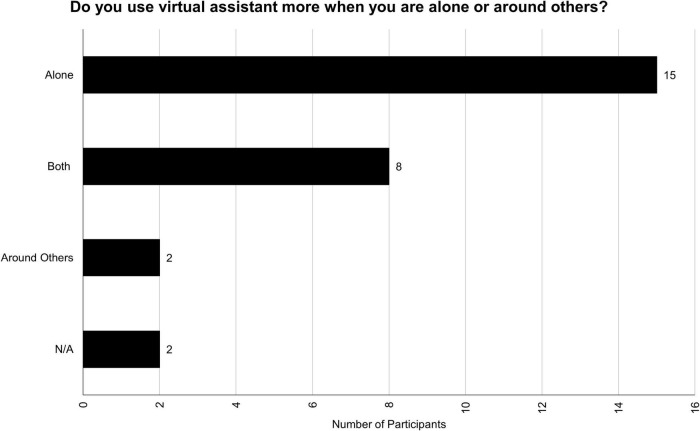
Do you use a virtual assistant more when you are alone (not around other people) or when you are around other people (with friends, on the train, shopping)?

One objective of our study was to investigate whether participants are more comfortable using their VAs when they are alone or when they are around others. Results show that, in general, participants prefer to use their VAs privately. The distribution for this theme can be found in Figure 3. For some of the participants, who prefer to use VAs alone, the reluctance of using them around other people is associated with awkwardness of talking to a technological device:

#1a: “I think it would be kind of weird if other people saw me just like getting my phone or just talking to it, you know?”

#8a: “I’m not really the kind of person to, like, ask my phone something in public, I think that’s strange…[the interviewer asked why is that] I don’t know. I don’t want to bother other people, and my mom does it and it’s weird…”

Ambient sounds and the need for quiet environments are other reasons why participants prefer to use VAs alone:

#11a: “Maybe it’s because of the ambient sound. When there is no disturbance, I can easily communicate with my virtual assistant. When there is [are] disturbing sounds, I have to shout at my phone. Hey, Google, Hey Google. So, I don’t want to do that in public…”

On the contrary, one of the users who prefers to use VAs around others mentions:

#25a: “I tend to use it the most when I’m with other people just because if we’re, like, in the middle of a conversation, I don’t want to have to stop and like manually type things in and look things up because I feel like it detracts from the conversation. I don’t want people to feel like I’m not paying attention to them. And so, in those cases, it’s a lot quicker to just say, hey, Siri, what are some restaurants near me or something like that.”

Some participants stated that they are comfortable with their VAs in both settings. These participants had no preference in which spaces they used their VAs:

#5a: “It doesn’t matter, actually. I don’t see it like that. Like if I’m getting used to it, I would use it in presence of anyone else.”

#6a: “I mean, I think I use it on both [occasions], when I’m alone as well as when I’m with my friends or the family. Yeah, it’s, it’s not really that it’s not really a question of when I’m using it. It just whatever a situation arises where I’m not able to use my hands and I don’t want to type something on my phone, I don’t want to unlock my phone. I can just say, it just goes, Hey Siri and get started on that.”

### Playfellow

**FIGURE 4:**
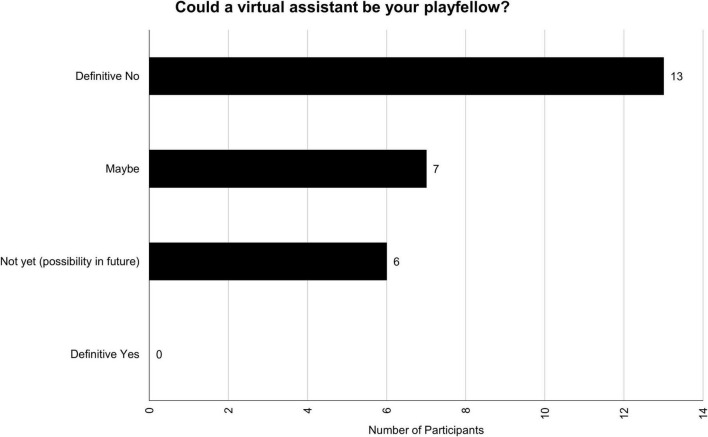
Could a virtual assistant be your playfellow?

Participants were asked “Could a virtual assistant be your playfellow (such as a friend, assistant, acquaintance, or partner)?” While it appears that some are undetermined, and some see it as a possibility, no one outright considered VAs as a playfellow. The distribution for this theme can be found in Figure 4. One participant, whose response was categorized as “maybe,” mentioned:

#8b: “Ummm, I think, I mean, I definitely see a big difference. I don’t know if I would call, like, my virtual assistant a friend, but definitely like, I feel friendly toward it.”

Another interviewee mentioned:

#4a: “Yes and no, I would say. I mean, it does help in some aspect, but you cannot rely on it [for] on everything.”

For the participants that did not consider VAs a playfellow in any way, one answered:

#26a: “Ah, I think at the deepest level I always look at it as a machine, even if it can answer back like Google, but not a play fellow. I mean, if we consider that situation it’s I’m trying to, I was trying to play with it, but not as a person.”

Similarly, another participant mentioned:

#10a: “Hmmm, no I don’t think so. I think I get really tired of it. Like, I can’t even play games with it for that long. I think it’s something about the robotic voice that kind of does it for me. Like, I know, they try to make it, like, more human. But still the way that it answers, there’s no like intonations or the way a human speaks is with you know, ups and downs. It’s more, like, flat. You know.”

A participant who thinks VAs could become playfellows in the future mentioned:

#18a: “I want it to be more like a human. I wish it was more like a human. And I want it to be a better assistant than it is, and I keep having this hopeful expectation. Every time I try to use it, that this time, it will give me the answer I need or want even though you know, it usually disappoints me. I keep trying because I do know that has gotten better over time. It’s just at a really ridiculously slow pace and feels like a ridiculously slow pace.”

### Companionship

**FIGURE 5:**
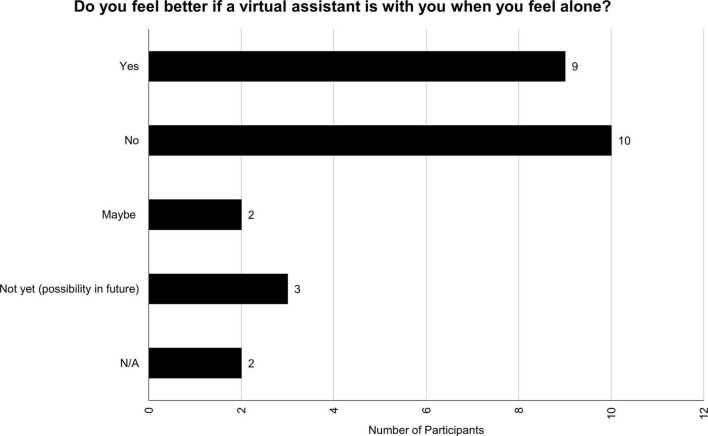
Do you feel better if a virtual assistant is with you when you feel alone?

When participants were asked whether they feel better if a VA is with them when they feel alone, the answers could be categorized into determined yes, determined no, maybe, and not yet (see Figure 5). One of the participants that considered VAs useful for feeling better when they feel lonely stated:

#6b: “There at least be a voice other than my own. So, I can like, even if it’s a meaningless conversation, I can still have that voice other than my own in the room.”

One participant who did not consider their VA as a companion, considered how VAs could exacerbate loneliness for them in this context:

#3a: “I think that would make me feel even lonelier. If I was feeling that lonely, I feel like I would always have somebody to talk to like another human being to talk to who I have a connection with. So, if I had to resort to the robot, it would mean that everyone has stopped being friends with me and my family no longer exists and there’s just no one out there for me other than complete strangers. And it would be the same as talking to one of the AIs as talking to a complete stranger.”

Others who found VAs helpful when they were alone, associated them with helping with boredom and safety:

#24a: “I feel better sometimes. When I get bored, and people are not around me. We get used to talk to virtual assistant sometimes, maybe we will ask for bedtime stories. It’s like narrating one night story and we’ll be like listening to it.”

#15a: “I feel like that comes with the phone too. I feel like it’s a really helpful tool. So yeah, I feel more safe [safer] when I have my phone. I feel like Wherever I am, I know how to get home. It has my contact so I can call someone for any help. So yeah, like the virtual assistant is just like an addition to what my phone provides.”

As a future possibility of companionship with a VA, a participant mentioned:

#18b “If it was sophisticated enough, that would be a wonderful thing to have. At a scale of 1–10, we’re at one and it would have to be 10 to be able to do that.”

### Trust

Participants were asked two questions regarding trust: “Do you trust the information you receive from your AI Virtual Assistant? Which information do you trust giving your Virtual Assistant?” These questions separate trust into two categories: receiving information and providing information. Regarding receiving information, while trust is not binary and can be a spectrum, the analysis of the transcriptions show that the answers can be general, with yes or no responses (18 out of 26) or can be categorized into simple and complicated information (6 out of 26). Two of the participants were former-users and were not considered in this theme.

Those participants who answered in a general yes or no manner, 16 out of 18 responded that they trust the information they receive from VAs. For instance, a participant who generally trusts VAs responded:

#22a: “Yeah, as if it [VA] like, tells me something back. As opposed to like a Google search, then like, I’ll trust it, like when it gives me an answer. Yeah, I’ll always try it because it’s like it’s from Google or whatever and so like, I trust [it].”

Another participant responded:

#4b: “Yeah. Yeah, definitely I trust it [VA]. I can’t say I trust it all the time but most of the times I think I trust it. Otherwise, I want to use it and I trust it because the information it gave back to me is also based on its search, like asking the weather, it would get information from my weather app. So, even if I don’t ask it, I will still go to that app and ask it and look at it myself. So, I think the way that we get information are basically the same. So, most of the time I trust it.”

In contrast, two participants do not generally trust VAs for receiving information. One participant mentioned:

#12b: “Trust? Sometimes, [interviewee changed their mind] no, no. It’s all internet, right? It’s not maybe, it [VA] doesn’t have to be always true. Whatever it is, it just goes to a Google or other internet platforms and gives you the feedback, that’s it.”

Six participants categorized their trust level into two groups of simple and complicated information. Those six participants all trust basic information (such as the weather and trivia) they receive from VAs, but they do not trust complicated information they receive like academic answers. One participant responded:

#26b: “Depends on the type of information I’m asking. If, for example, when I’m in the car, I just tell it [VA] [to] navigate me to home. I don’t even look it up because it [VA] knows where my home is and it has GPS and knows better, you know, [than] what I know about my environment. So, I trust it. But, if it gets to the more complicated ones, I think I never use it. I don’t even trust it that much to even use it for more complicated stuff.”

Another participant responded:

#2a: “I trust the answers to simple questions. Is it going to rain today? or What’s the name of this song? I don’t trust a lot of other information that might come out of virtual assistants.”

Participants’ responses to “Which information do you trust giving your virtual assistant?” were all related to personal information that could be categorized into personal confidential and personal non-confidential information. Out of 26 participants, 15 would only share their personal non-confidential information. A participant mentioned:

#6c: “Um, well, as far as I know, I think I’ve never been asked for any particular information from my virtual assistant. So, I would know that, but if they [VAs] will ask questions, I would definitely be open to sharing personal information that wouldn’t be a problem because they [VAs] have a good, very good confidentiality clause. When in the use of [using] Siri on a daily basis for suggestions and for all of these predictions, all of these simple tasks that we use Siri for on a regular basis, so that’s not really a problem for me. I would share my personal information. I mean, yeah.”

Some participants are not comfortable sharing any type of personal information:

#10b: “No, no. It’s just data. So, data is so important and so valuable these days, like your personal data. So, that’s why I don’t like [to] connect any commercial accounts or anything, or actually my credit card or bank information like, not to the Google Home because anyone can kind of use that. It’s very like low security. So, I wouldn’t give it that kind of information. Like, I wouldn’t say Hey Alexa, order me whatever, you know, from Amazon. One other reason for that, like not for ordering stuff from Amazon or Google or whatever, would be that I actually prefer looking at all my options before I buy something. So, I wouldn’t just trust its choice in getting any object that I want.”

### Emotional attachment

Most participants (18 out of 26) did not report any emotional attachment to their VAs. A correlation was identified between not forming emotional attachment to VAs and a lack of human-like attributes. One participant did not report an emotional attachment due to the lack of empathy and agency in VAs:

#22b: “Attachment? No, not really. Because, as I said, I don’t think there’s any emotion, you know, it’s just, like, they’re to answer your questions, rather than it being like, its own, like, entity. So, I don’t think of it as, like a person, you know, it’s just, it’s just some like a tool to answer questions.”

Similarly, for another participant, it is the lack of empathy, agency, and interactivity that prevents the formation of emotional attachment:

#15a: “It’s very one sided, what the relationship is like. I can ask it and it will respond. But that’s it. Like I can never just be walking around and ask Hey, how am I doing? It cannot, like, check up on me when I’m feeling sad. I can’t, like, say: hey let’s go out to eat, just have fun. We can’t go to the park so it’s like it’s very, I don’t know the right word, one sided, boring, very static relationship.”

One participant identified that if VAs had more human-like features, emotional attachments may be possible in the future:

#13a: “I think possibly like as we progress with the tech and it becomes more smoothly integrated into our lifestyle there could be like, starts and like thoughts on that, like, if you were to drop like Alexa into like a robot or something and give it like this, I guess human like characteristics and it’s like responding to your actions and whatnot, then we can, like, maybe start to get down that path of like, like deciding, like, that whole debate over like robotics ethics if they’re big becoming aware, as our virtual assistants get more intelligent. I think later down the line that’s probably like a topic that we will eventually start crossing.”

Some participants identified distinctions between attachments to their smartphones and attachments to the VA technology:

#15b: “I’m attached to my phone because I use it for a lot of things, but the virtual assistant itself no…”

Another participant states:

#17a: “I feel attached to my phone, which has a virtual assistant on it. I wouldn’t say I feel attached specifically to the virtual assistant.”

### Comparison with human beings

Although our interview questions were not designed to directly assess the comparison of VAs with human beings, based on analyzing the entirety of interview transcripts, we identified that participants tend to compare features of VAs with human beings in their responses, especially when they were asked about advantage/disadvantages, loneliness, playfellow, emotional attachments, and disclosure categories of the codebook in Table 1. Some participants compared VAs with humans without ascribing humanlike characteristics while some did attribute humanlike characteristics to VAs, those who did, used gendered pronouns (she/he, her/him, hers/his) to address VAs. A total of 9 participants used these pronouns 56 times collectively. An example of such a response can be found in the following:

#18b: “The fact that there is a voice in my phone that speaks and responds to a question at all, is still, has an air of magic. Even doing a simple thing like when she does. see I even said she like she’s a person. When she does not fail. When she succeeds rather at providing the information, it really does feel like some kind of magic has happened.”

**FIGURE 6:**
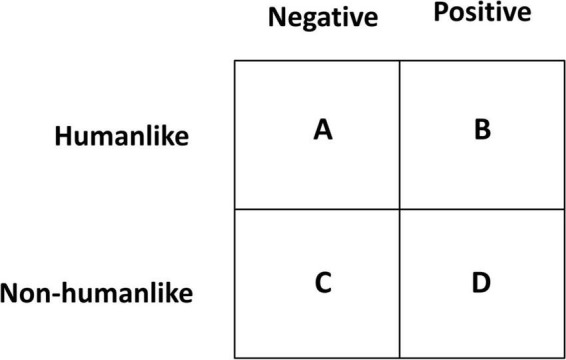
Characteristics of VAs compared with human beings.

The interviews illustrate that, compared to human beings, VAs have both humanlike and non-humanlike characteristics. Furthermore, each characteristic (either similar or different from human beings) can also be positively or negatively perceived (see Figure 6). A notable set of characteristics are those that are both non-humanlike and also positively perceived [such as being unable to hurt others (Figure 6, Box D)]. The existence of such characteristics is helpful in revealing that humanlike characteristics are not the only group of features that can result in increased interaction with VAs. There are characteristics exclusive to VAs that can also increase interaction.

A feature similar in human beings and VAs with a positive bearing is a reassuring voice (Figure 6, Box B):

#10c: “I mean just to have, like, someone like talkback to me. If I say something like that would give me a sense of kind of that I’m not alone. So, yeah in that way it [VAs] would help.”

#10d: “Especially if you’re, like, in an empty hall, you know, in your apartment or your house, you know, you just, like, hear another human being’s voice [interviewee is referring to VA’s voice as human being’s voice].”

Not all similarities have a positive bearing. Leaking information in the form of sharing conversations is a feature that one interviewee considered similar between humans and VAs but with a negative bearing (Figure 6, Box A):

#3b: “They [VAs] leak about as much as we would expect a normal human to leak, but an AI doesn’t know what, what information is the most important and what they absolutely shouldn’t let go.”

There were also characteristics mentioned that detailed a negative conception of the non-humanlike traits of VAs (Figure 6, Box C):

#11b: “It’s not a human being. It doesn’t have any feelings.”

#4c: “But it’s never, like, it can sense from my tone. Oh, you’re not happy now or you’re sad or you’re excited. It definitely cannot sense that.”

Some interviewees mentioned notable VA characteristics that are different from human beings but have a positive bearing. An example of such characteristics is trust in machines and their inability to be hurtful (Figure 6, Box D):

#14a: “If you see a human being, we cannot trust the human completely. Here’s the thing, you can trust Alexa, of course it’s a non-living object and it’s not going to hurt you.”

#21a: “Sometimes, I’ve had thoughts like machines, they are a lot more confidential. Like if you tell people they can tell other people and like sometimes you want things to be a secret. So, it’s, like, easier to tell, like a machine that won’t tell anyone.”

While it is it not our intention to promote anthropomorphism in scientific research as Salles et al. (2020), p. 94) mention, “it would be both misleading and risky not to make efforts to raise awareness of and limit its manifestations, especially within the scientific community,” when discussing VAs and making sense of VAs, comparing some features of VAs to human characteristics is common practice. Regarding empathy, one participant mentioned:

#20a: “I feel like I would probably talk to a human being more, because of the empathetic feel, because they’re [human beings] able to relate. But again, the Google Home is a machine.”

Regarding memory, another participant mentioned:

#10e: “I mean, having a real conversation with someone, you know, that person would remember that I said, I was happy. it [VAs] does not remember.”

Regarding agency, a participant asserted:

#25b: “I think in order to develop a relationship with AI, you have to reach the point where you see it as like an independent entity. And that’s something that you can connect with.”

Several participants clearly had some anthropomorphizing tendency of interacting with or perceiving their VA. One participant mentioned calling their VA names or cursing at it:

#2b: “No, I call it names when it’s not working correctly. [(.)] It depends on the day. Sometimes I compare it to fun historical figures and I tell it it’s a failure like Napoleon. Sometimes I just curse at it and call it an idiotic piece of crap. It really depends. And then sometimes I give it a compliment, because it actually figured out what I wanted for once.”

One participant characterized their VA as a conversational partner:

#12a: “Yeah, it’s a good listener…And you don’t need its opinion.”

Another participant compared his VA to his girlfriend:

#14b: “Sometimes if you’re talking to Alexa and sometimes you feel like your girlfriend or friend. My friend’s name is [(name)]. So sometimes the picture of [(name)] comes to mind while I talk to Alexa.”

We identified that 9 participants used gendered pronouns to address VAs, considered as a way of personifying VAs. A participant mentioned:

#25c: “I think she is responsive to my commands. But I don’t like I just I don’t generally share my feelings with Siri I don’t know if she has the capability to properly respond to that, you know, at the end of the day I don’t think AI can replace humans ever.”

## Discussion

Based on the results, it appears that in the current state of VAs, participants employ them in a basic manner for performing simple tasks. Similarly, the same notion is observed regarding the matter of trust in both receiving and providing information (i.e., participants trust VAs for basic information). The fact that most participants prefer to use their VAs in private, reveals that they do not perceive interaction with VAs in public as socially acceptable. Most participants said they do not consider VAs to be playfellows nor perceived them as companions helping them with feelings of loneliness. We identified the important role of recognizing humanlike characteristics that shape user perceptions of VAs, both for the majority who did not perceive VAs as their playfellow or companion and those who did. These results can be seen against the background of research that shows that in the context of human-technology communication, when interacting with communicators that belong to a different ontological category, people’s conceptualizations of the nature of humans and machines play a role of how they understand and interact with the technology (Guzman, 2020).

As asserted by Chérif and Lemoine (2019), anthropomorphic characteristics can cause attachment in some users. In our analysis, while it was not within our original research goals to highlight the anthropomorphic features of VAs from a methodological perspective, we could not help but notice that several participants compare and contrast the capabilities of VAs to human capabilities, specifically human mental capabilities such as empathy, language/voice, memory, agency, and interactivity. On the other hand, Anthropomorphism can be problematic as we might become prone to equating AI features with that of the human mind, which may negatively impact and limit the ethical analysis of issues related to AI (Salles et al., 2020).

Like the work by Pradhan et al. (2019), we identified that 9 of the participants used gendered pronouns to personify VAs. This finding mirrors work by Gao et al. (2018), who performed content analysis based on reviews of Amazon Echo and identified personification of Alexa. One review stated:

“Please let your wife know about her beforehand, or there will be hell to pay. She might think you are having an affair because you are spending more time talking to Alexa than her” (Gao et al., 2018, p. 8).

Based on our results, it is likely that, in practice, conscious emotional attachment to VAs is less exaggerated. While more empirical research is needed to find out users’ tendency to form emotional attachment with technology, and to ascribe human-like behavior to technology, it is equally important to critically analyze claims that allude to widespread anthropomorphizing. In our study it appears that participants assess their relationship with VAs based on the VA having, or not having, certain properties or capabilities rather than focusing on what takes place on relational levels.

What we do find is a confirmation of an attachment to smartphones for meeting personal needs. Such a form of attachment is aligned with work by Fullwood et al. (2017) in which they approached attachment to smartphones through the lens of Uses and Gratifications theory. Within answers to questions regarding emotional attachment to VAs in our interview questions, “attachments” were not described as emotional attachments but rather as attachments to the conveniences of the technology, attachments in the form of dependence on the technology to perform various tasks for the individual.

Our study did not directly ask participants about questions of autonomy or delve into automaticity theories regarding VAs (Bauer and Dubljević, 2020). Thus, from our study, we cannot claim that VAs directly or indirectly influence autonomy or automaticity. However, what is notable from our findings, is that participants are not using VAs for arduous tasks, rather, simple tasks such as setting alarms or timers, playing music, asking for directions, and scheduling appointments.

The comparison with human beings theme illustrates that participants expect VA attributes to be more humanlike in the future, which may increase the probability of forming a deeper relationship, such as emotional attachment. Participant accounts reflected that at the current state, it is the lack of human-like capabilities of VAs that prevents participants from forming a deeper relationship such as emotional attachment with them. Our study did not reveal emotional attachment to VAs among the study participants.

### Study limitations

- Participants are students at a technical university and could be considered technology savvy users. Several interviewees stressed the technological nature of VAs. This is in contrast to other research (Pradhan et al., 2019) with a group of older adults.
- VAs have not reached a technical level required for users to develop emotional bonds with the technology. However, in other studies that stress emotional involvement, relatively simple technology had been used (Bartneck and Hu, 2008; Darling et al., 2015).
- Our results represent what participants disclosed, what came to their minds during the interview, what they were aware of, or what they were willing to share. It could be argued whether a more direct approach, using experiments, would reveal more emotional attachment, social, and natural responses to media and other forms of technology that are not consciously disclosed (Reeves and Nass, 2002).
- Cultural factors could play a role in emotional attachment to VAs.

## Conclusion

By conducting 26 episodic interviews focusing on the perceptions of VAs, analyzing the transcriptions by performing content analysis according to the abductive analysis method, and comparing the findings with available theoretical frameworks, we have identified how participants perceive VAs based on seven themes: features and tasks, spatial awareness, playfellow, companionship, trust, perceived emotional attachment, and comparison with human beings.

Our findings show that, currently, participants use VAs for simple tasks such as playing songs and setting reminders that otherwise require manual labor. Participants prefer to use their VAs in private, due to current socially acceptable norms. In their current state, and compared with human beings, while VAs have some positive non-human characteristics, it is the conceived lack of human-like capabilities that prevents participants from perceiving VAs as playfellows, companions, and forming an emotional attachment to them.

For future work, it will be important to conduct more empirical research on human-technology interaction and on participants’ tendency to ascribe human-like behavior to technology and forming emotional attachment with technology. Human-technology interaction is constantly evolving, influenced by a broad spectrum of factors. It would be interesting to investigate the impact of isolation caused by the Covid-19 pandemic as it may have affected the perception of VAs.

## Data availability statement

The datasets analyzed for this study are not publicly available due to privacy or ethical restrictions. Queries regarding the datasets should be directed to EH, ehildt@iit.edu.

## Ethics statement

The study involving human participants was reviewed and approved by the IRB, The Illinois Institute of Technology. The participants provided their written informed consent to participate in this study.

## Author contributions

LM, MS, and EH planned the study, analyzed the interviews, and wrote the manuscript. LM and MS conducted the interviews. All authors contributed to the article and approved the submitted version.

## Footnotes

1 2013, directed by Spike Jonze; United States: Warner Bros. Entertainment Inc.

2 2019, directed by Jon Lucas and Scott Moore; United States: Lionsgate.

3 Available at: https://youtu.be/iNxvsxU2rJE; and https://youtu.be/xxNxqveseyI.

## Appendix

### Interview questions for frequent users and former users

**Frequent users**

General:

1. Do you use virtual assistants like Siri, Google Assistant and Alexa? If yes, which ones do you use?
2. How often do you use your Virtual Assistant? Which one do you use most?
3. For what simple functions do you use it (like setting an alarm, checking the weather, etc.).
4. How does it assist you for more complicated tasks? (beyond searching the internet)
5. What makes it special for you?

Communicational:

1. Is it easy to communicate and interact with your Virtual Assistant?
2. Are there language barriers when you use your Virtual Assistant?
3. What are the advantages of communicating with a Virtual Assistant over physically typing or communicating with human beings?
4. Do you use a Virtual Assistant more when you are alone (not around other people) or when you are around other people (with friends, on the train, shopping)?
5. Do you think Virtual Assistants can understand you?
6. Do you trust the information you receive from your AI Virtual Assistant? Which information do you trust giving your Virtual Assistant? (What kind of trust? Trust in which context?).

Emotional:

1. Do you think your Virtual Assistant is aware of and responsive to your intentions, actions, and feelings?
2. Could a Virtual Assistant be your playfellow (such as friend, assistant, acquaintance, or partner)?
3. Do you feel better if a Virtual Assistant is with you when you feel alone? How so?
4. Would you disclose more to a Virtual Assistant rather than a human being?
5. Do you form attachments to your virtual assistant? If no, why not? If yes, What kind of attachments?
6. Do you establish a relationship with it? If yes, what kind of relationship? If no, what prevents you from establishing a relationship?
7. How do you define a relationship?

Demographics:

1. What gender do you identify with?
2. How old are you?

What is your native language?

1. How long have you been in the United States?

**Former users**

General:

1. Do you use Virtual Assistants like Siri, Google Assistant and Alexa? If no, what else do you use?
2. Would you ever use a Virtual Assistant?
3. Do you think it is strange to use a Virtual Assistant?
4. Do you think using these kinds of Virtual Assistants affect people’s lives? If yes, how might it affect people’s lives?
5. Are you worried about using a Virtual Assistant? Why or why not?

Communicational:

1. Do you find it hard to communicate with Virtual Assistants? If yes, what makes it difficult?
2. Since you do not use Virtual Assistant, do you have negative concerns regarding Virtual Assistants in your mind that prevent you from using it?
3. Is there a language barrier that prevents you from using Virtual Assistants? (For international students).
4. Would you use Virtual Assistants if it was available in your language? (For international students)

Emotional:

1. Do you think people react emotionally to a Virtual Assistant? If so, what sort of emotions? If not, why not?
2. Do you think it is possible to form an emotional attachment with a Virtual Assistant similar to the bond with pets or even humans? If yes, how so? If no, why not?

Demographics:

1. What gender do you identify with?
2. How old are you?
3. What is your native language?
4. How long have you been in the United States?

## References

[B1] Bartneck C. Hu J. (2008). Exploring the abuse of robots. *Interact. Stud.* 9 415–433. 10.1075/is.9.3.04bar 33486653

[B2] Bauer M. W. (2000). “Classical Content Analysis: a Review,” in *Qualitative Researching With Text, Image And Sound: A Practical Handbook*, eds Atkinson P. Bauer M. W. Gaskell G. (Thousand Oaks: SAGE Publications). 10.4135/9781849209731.n8

[B3] Bauer W. A. Dubljević V. (2020). AI assistants and the paradox of internal automaticity. *Neuroethics* 13 303–310. 10.1007/s12152-019-09423-6

[B4] Chérif E. Lemoine J.-F. (2019). Anthropomorphic virtual assistants and the reactions of Internet users: An experiment on the assistant’s voice. *Rech. Appl. Mark.* 34 28–47. 10.1177/2051570719829432

[B5] Coeckelbergh M. (2018). How to describe and evaluate “deception” phenomena: Recasting the metaphysics, ethics, and politics of ICTS in terms of magic and performance and taking a relational and narrative turn. *Ethics Inf. Technol.* 20 71–85. 10.1007/s10676-017-9441-5

[B6] Cooper R. S. McElroy J. F. Rolandi W. Sanders D. Ulmer R. M. Peebles E. (2008). *Personal Virtual Assistant.* U.S. Patent No: 6,757,362. Durham, NC: Avaya Inc.

[B7] Cooper R. S. Sanders D. Ulmer R. M. (2011). *Personal Virtual Assistant.* Google Patent No: US 8,000,453 B2. Basking Ridge, NJ: Avaya Inc.

[B8] Danaher J. (2018). Toward an ethics of ai assistants: An initial framework. *Philos. Technol.* 31 629–653. 10.1007/s13347-018-0317-3

[B9] Danaher J. (2020). Welcoming robots into the moral circle: A defence of ethical behaviourism. *Sci. Eng. Ethics* 26 2023–2049. 10.1007/s11948-019-00119-x 31222612

[B10] Darling K. (2016). “Extending legal protection to social robots: The effects of anthropomorphism, empathy, and violent behavior towards robotic objects,” in *Robot Law*, eds Ryan C. Michael A. F. Ian K. (Cheltenham: Edward Elgar Publishing).

[B11] Darling K. Nandy P. Breazeal C. (2015). “Empathic concern and the effect of stories in human-robot interaction,” in *2015 24th Ieee International Symposium On Robot And Human Interactive Communication (Ro-Man)*, (Piscataway: IEEE), 770–775.

[B12] Flick U. (2000). “Episodic Interviewing,” in *Qualitative Researching With Text, Image And Sound: A Practical Handbook*, eds Atkinson P. Bauer M. W. Gaskell G. (Thousand Oaks: SAGE). 10.4135/9781849209731.n5

[B13] Frey L. Botan C. H. Kreps G. (2000). *Investigating Communication: An Introduction To Research Methods.* Boston: Allyn & Bacon.

[B14] Fullwood C. Quinn S. Kaye L. K. Redding C. (2017). My virtual friend: A qualitative analysis of the attitudes and experiences of Smartphone users: Implications for Smartphone attachment. *Comput. Hum. Behav.* 75 347–355. 10.1016/j.chb.2017.05.029

[B15] Gambino A. Fox J. Ratan R. A. (2020). Building a stronger CASA: Extending the computers are social actors paradigm. *Hum. Mach. Commun.* 1 71–85. 10.30658/hmc.1.5 34740688

[B16] Gao Y. Pan Z. Wang H. Chen G. (2018). “Alexa, my love: Analyzing reviews of amazon echo,” in *2018 IEEE SmartWorld, Ubiquitous Intelligence & Computing, Advanced & Trusted Computing, Scalable Computing & Communications, Cloud & Big Data Computing, Internet of People and Smart City Innovation (SmartWorld/SCALCOM/UIC/ATC/CBDCOM/IOP/SCI)*, (Piscataway: IEEE), 372–380. 10.1109/SmartWorld.2018.00094

[B17] Gunkel D. J. (2018). The other question: Can and should robots have rights? *Ethics Inf. Technol.* 20 87–99. 10.1007/s10676-017-9442-4

[B18] Gunkel D. J. (2020). *An Introduction To Communication And Artificial Intelligence.* Cambridge: Polity Press.

[B19] Guzman A. L. (2019). Voices in and of the machine: Source orientation toward mobile virtual assistants. *Comput. Hum. Behav.* 90 343–350. 10.1016/j.chb.2018.08.009

[B20] Guzman A. L. (2020). Ontological boundaries between humans and computers and the implications for human-machine communication. *Hum. Mach. Commun.* 1 37–54. 10.30658/hmc.1.3

[B21] Hirschberg J. Manning C. D. (2015). Advances in natural language processing. *Science* 349 261–266. 10.1126/science.aaa8685 26185244

[B22] Koike M. Loughnan S. (2021). Virtual relationships: Anthropomorphism in the digital age. *Soc. Pers. Psychol. Compass* 15:e12603. 10.1111/spc3.12603

[B23] Norouzi N. Kim K. Bruder G. Erickson A. Choudhary Z. Li Y. (2020). “A systematic literature review of embodied augmented reality agents in head-mounted display environments,” in *Proceedings of the International Conference on Artificial Reality and Telexistence & Eurographics Symposium on Virtual Environments*, eds Argelaguet F. Sugimoto M. McMahan R. (Orlando, FL: University of Central Florida).

[B24] Norouzi N. Kim K. Hochreiter J. Lee M. Daher S. Bruder G. (2018). “A systematic survey of 15 years of user studies published in the intelligent virtual agents conference,” in *Proceedings Of The 18th International Conference On Intelligent Virtual Agents*, (Sydney), 17–22. 10.1145/3267851.3267901

[B25] Pradhan A. Findlater L. Lazar A. (2019). Phantom Friend” or “Just a Box with Information” Personification and Ontological Categorization of Smart Speaker-based Voice Assistants by Older Adults. *Proc. ACM Hum. Comput. Interact.* 3 1–21. 10.1145/335931634322658

[B26] Reeves B. Nass C. I. (2002). *The Media Equation: How People Treat Computers, Television, And New Media Like Real People And Places.* Cambridge: Cambridge university press.

[B27] Salles A. Evers K. Farisco M. (2020). Anthropomorphism in AI. *AJOB Neurosci.* 11 88–95. 10.1080/21507740.2020.1740350 32228388

[B28] Scheutz M. (2011). “The Inherent Dangers of Unidirectional Emotional Bonds between Humans and Social Robots,” in *Robot Ethics: The Ethical And Social Implications Of Robotics*, eds Patrick L. Abney K. Bekey G. A. (Cambridge: MIT Press).

[B29] Timmermans S. Tavory I. (2012). Theory construction in qualitative research: From grounded theory to abductive analysis. *Sociol. Theory* 30 167–186. 10.1177/0735275112457914

